# The development of a scoring and ranking strategy for a patient-tailored adverse drug reaction prediction in polypharmacy

**DOI:** 10.1038/s41598-020-66611-8

**Published:** 2020-06-12

**Authors:** Andrei Valeanu, Cristian Damian, Cristina Daniela Marineci, Simona Negres

**Affiliations:** 1 Carol Davila University of Medicine and Pharmacy, Faculty of Pharmacy, Department of Pharmacology and Clinical Pharmacy, 6 Traian Vuia St., 020956 Bucharest, Romania; 2 Polytechnic University, CEOSpaceTech, 7 Gheorghe Polizu St., Building P, 1st. floor, 011061 Bucharest, Romania

**Keywords:** Computational biology and bioinformatics, Health care, Medical research, Risk factors, Mathematics and computing

## Abstract

Only few applications are currently dealing with personalized adverse drug reactions (ADRs) prediction in case of polypharmacy. The study aimed to develop a patient-tailored ADR web application, considering characteristics from 734 drugs and relevant patient related factors. The application was designed in Python using a scoring and ranking system based on frequency and severity, computed for each ADR and expressed through an online platform. A neural networks algorithm was used for predicting the severity of ADRs. The application inputs are: age, gender, drugs, relevant pathologies. The outputs are: an overall severity profile (hospitalization and mortality risk), a stratified risk on specific ADR groups and a sorted list of the most important ADRs depending on frequency and severity. The Severity prediction model validation resulted in 79.7–85.1% Area Under the Receiver Operating Characteristic Curve Score, which lies in the good cut-off of 75–90%. The program offers a complex view regarding the ADR profile of a given patient and could be used by the physician and clinical pharmacist during patient safety monitoring, for a coherent therapy choice or medication adjustment, due to the good therapy coverage and the inclusion of relevant patient comorbidities.

## Introduction

Currently, adverse drug reactions (ADRs) represent major worldwide health issues, due to their negative impact on the quality of life and outcome of patients receiving multiple drug therapy. It is believed that almost 10% of the in-patients usually develop at least one ADR during hospitalization, while almost 50% of all ADRs appear prior to the admission^1–5^. The major risk factors which specifically influence the development of ADRs are age, gender, ethnicity, pregnancy, multiple pathology, polypharmacy, drug dosage and frequency of administration, genetic polymorphism or special conditions (renal impairment, hepatic impairment)^6–9^.

The complex combination of these factors leads to the need of implementing precision medicine in case of a drug therapy for a certain patient^10^. Therefore, the consideration of a patient-tailored ADR profile becomes of an utmost importance when choosing the best drug combination and during the patient safety monitoring.

Several standardized databases containing valuable information regarding ADRs can be found, which could aid in extracting the information for such purposes. Among them, PROTECT ADR database lists all the ADRs and additional information derived from the Summaries of Product Characteristics of a high number of centrally authorized products by the European Medicines Agency^11^.

A prediction of the ADRs which are likely to appear for a certain patient considered pre-clinical data and spontaneous reports and was described by Ngufor and Wojtusiak^12^, which took into account the specific drug information and known ADRs from DrugBank and Side Effect Resource Database (SIDER), as well as drug-event signals and demographics from Food and Drug Administration Adverse Event Reporting System (FAERS) and MedEffect. In terms of personalized tools, the American College of Cardiology launched an online application measuring Statin intolerance, taking into account specific patient and drug information. It provides a very complex analysis in terms of musculoskeletal ADRs for patients receiving statin therapy^13^. Drugs.com Interactions Checker is also a valuable and clinically relevant online tool in terms of assessing the drug interaction severity^14^. Other works mainly focused on ADR severity ranking^15^, while other recent publications estimated the hospitalization risk due to ADRs in elderly patients^16^.

However, the low number of online resources available for building personalized adverse drug reaction (ADR) profiles makes very useful the development of more medical applications to help the healthcare professionals in the ADR monitoring and therapy choice or therapy adjustment. In our study, we developed in Python programming language such an application, with emphasis on the clinically relevant factors which could influence the ADR severity in terms of hospitalization risk and mortality. The application included several graphical and risk profiles for a given patient and was also based on a neural networks severity prediction model validated by using relevant statistical measures. The novelty of our work consists in the patient-tailored stratification of individual ADRs for a given drug therapy, reflected in the scoring and ranking strategy which allows the creation of easily interpretable personalized ADR profiles.

Therefore, the aim of the present study was to develop a patient-tailored ADR computer application prototype, with a friendly graphical user interface (GUI), embedded in an online platform, which should be easily used by physicians and pharmacists. The application is based on a personalized ADR prediction in case of polypharmacy by the implementation of a scoring and ranking strategy.

## Results

### Example of implemented ADR application

**Figure 1:**
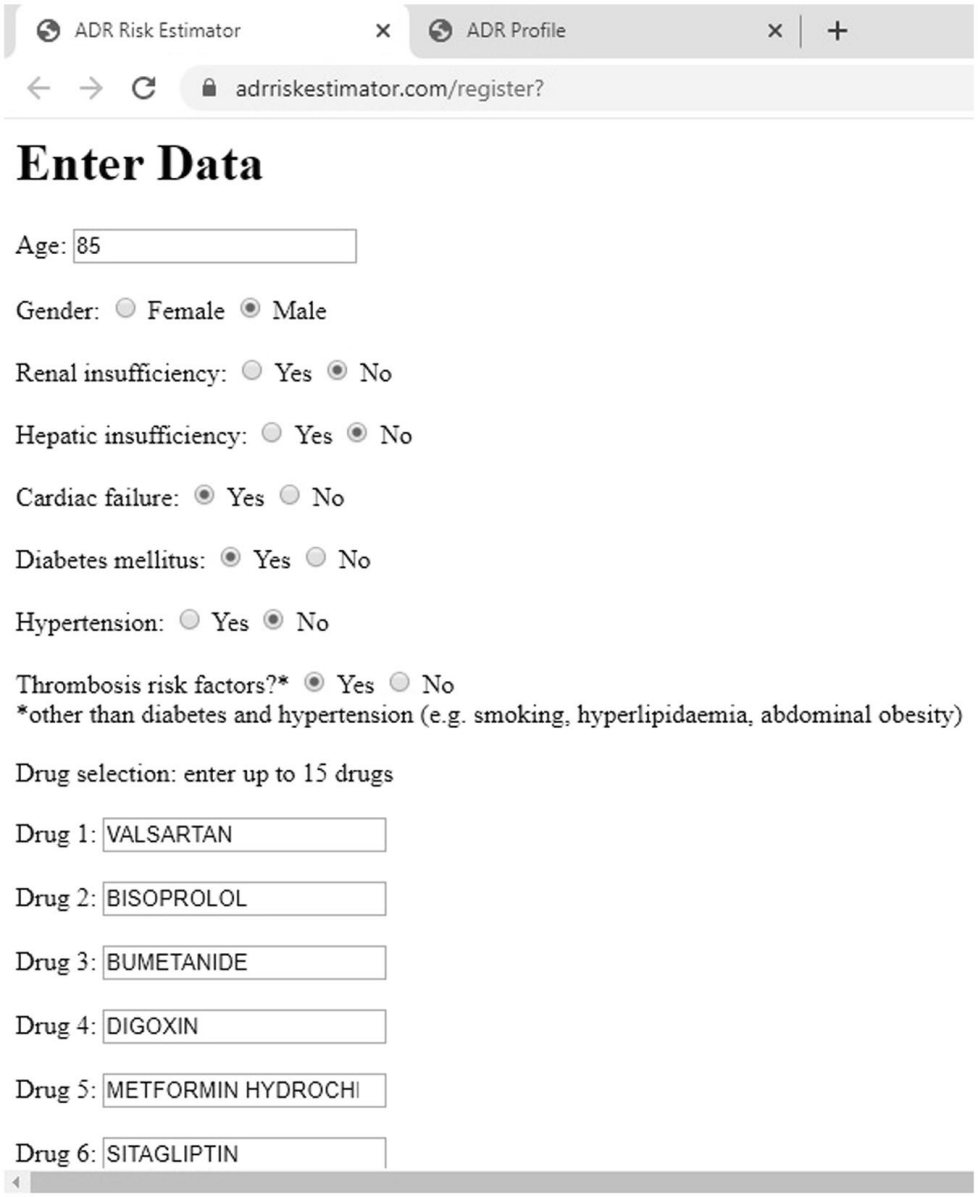
The online user interface for entering the inputs; after selecting each field, the program will use the inputs for computing the ADR scores.

Figure 1 presents the Graphical User Interface for entering the patient data which is embedded in the online platform.

**Figure 2:**
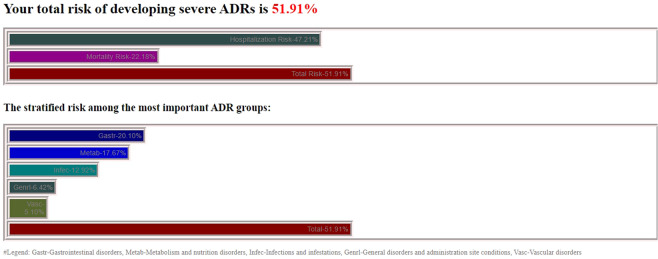
Plot 1: The Total Score (51.21%) (as well as the Hospitalization Risk and Mortality Risk). Plot 2: The SOC plot, obtained for the presented case for the first 5 SOCs in terms of severe ADRs: Gastrointestinal disorders, Metabolism and nutrition disorders, Infections and infestations, General disorders and administration site conditions, Vascular disorders.

**Figure 3:**
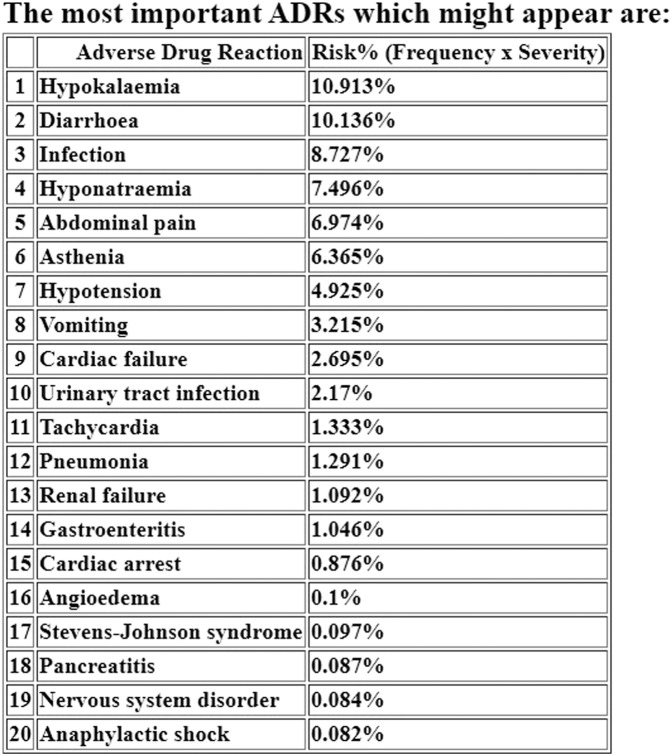
The sorted ADR list, containing the most relevant 20 ADRs in terms of combined frequency and severity for the presented case.

Figures 2 and 3 present an overall hospitalization and mortality risk estimation, a SOC plot and the list containing the most relevant ADRs in terms of severity which were returned for a complex patient case from FAERS; the 85-year-old male patient received a multi-drug therapy with valsartan, bisoprolol, bumetanide, digoxin, metformin, sitagliptin, gliclazide and simvastatin and suffered from 3 relevant pathologies: Cardiac failure, Diabetes mellitus and Hyperlipidaemia (thrombosis risk factor). The patient developed vomiting, renal failure and septic shock, was hospitalized and died due to the adverse drug reactions. The prescription information was analyzed and no patient counselling was involved.

### Severity score validation

**Figure 4:**
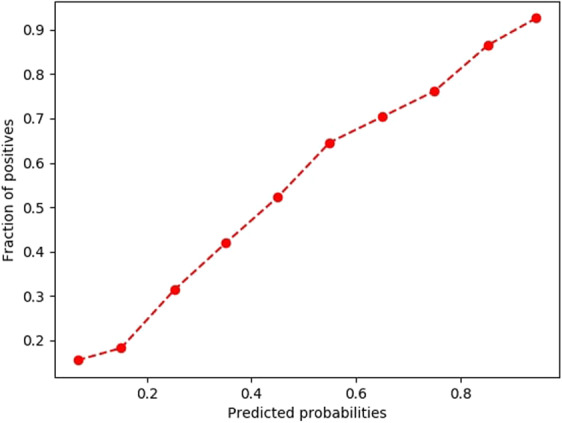
The reliability curve obtained for the validation of the Hospitalization prediction model.

**Figure 5:**
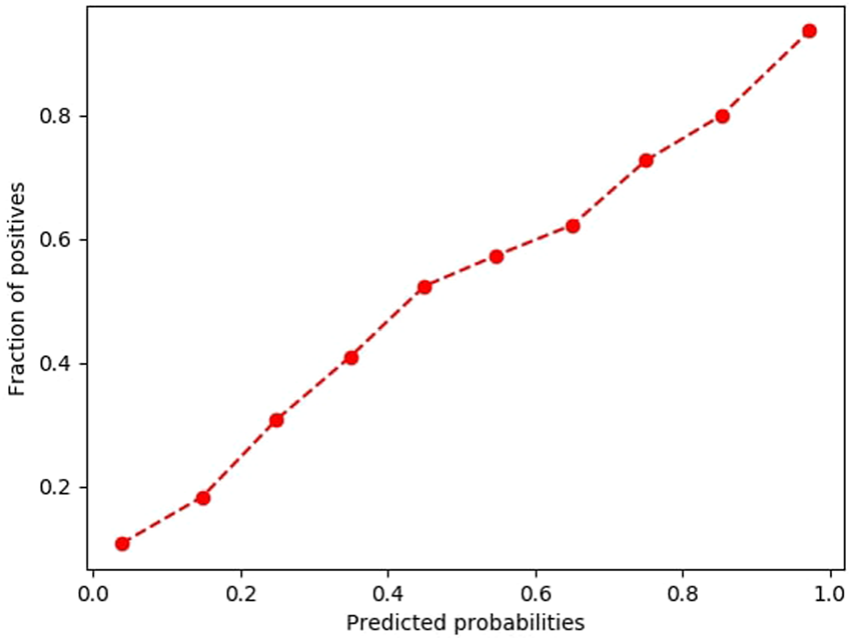
The reliability curve obtained for the validation of the Mortality prediction model.

Table 1 briefly presents the results obtained for the Severity Score validation, taking into consideration the computed measures. Figures 4 and 5 present the graphical representations of the fraction of positives (belonging to class 1 and hence being hospitalized or fatal cases) as a function of the predicted probability – the two figures illustrate how reliable the probabilities estimated by the neural networks model are.

**Table 1:** Validation results of the 4 machine learning classifiers*.

Validation measure	Algorithm**			
Hospitalization class prediction	MLP^#^	LR	RFC	KNN
Accuracy	72.23%	68.53%	70.94%	54.39%
Precision	78.57%	77.37%	77.60%	76.91%
Recall	73.01%	66.22%	71.57%	32.82%
F1 Score	75.69%	71.36%	74.46%	46.00%
**Hospitalization probability prediction**	**MLP**^**#**^	**LR**	**RFC**	**KNN**
Brier Score	18.43%	20.29%	18.98%	25.84%
ROC AUC Score***	79.17%	75.16%	78.16%	64.68%
**Mortality class prediction**	**MLP**^**#**^	**LR**	**RFC**	**KNN**
Accuracy	76.74%	73.32%	70.13%	62.34%
Precision	79.58%	75.47%	92.67%	60.76%
Recall	71.95%	69.09%	43.73%	69.69%
F1 Score	75.57%	72.14%	59.42%	64.92%
**Mortality probability prediction**	**MLP**^**#**^	**LR**	**RFC**	**KNN**
Brier Score	15.76%	18.76%	19.71%	23.01%
ROC AUC Score***	85.40%	81.26%	85.21%	68.74%

*Values taking into consideration a hold-out validation by splitting the data into training set – 65694 patients and test set – 16987 patients; **MLP = Multi-Layer Perceptron Classifier, LR = Logistic Regression, RFC = Random Forest Classifier, KNN = K-Nearest Neighbors; ***Most important validation measure; ^#^MLP was chosen as the algorithm for the application implementation due to the best results in terms of Severity Score validation

## Discussion

The ADR computer application prototype presented in this paper is designed to be a personalized predictive model able to sort, based on a specific scoring and ranking system, the most relevant ADRs which are likely to appear for a certain patient in case of multiple pathologies and polypharmacy.

Both score types (Frequency and Severity) are computed based on a probabilistic approach. The choice of combining them into a total score for each ADR was considered appropriate, since a combined score on each of the most severe ADRs would highlight the most clinically relevant adverse events. In addition, the final computation of a Total Score reflecting the patient risk was considered a useful option, since it could be used for patient stratification and therapy comparison/therapy choice.

The personalized ADR application is able to retrieve and organize ADR lists from well-known databases^17–19^.

Several scoring systems which work on specific ADRs are already documented in the medical literature^20,21^, measuring the causality or severity; however, they judge certain cases with ADRs which were already experienced and therefore could not work as a predictive tool before starting the drug therapy. Another study aimed at developing a prediction of hospitalization risk due to ADRs (the PADR-EC Score); relevant patient characteristics were taken into account, with a reliable validation process^16^. Nonetheless, the score was defined per patient and did not involve any ADR stratification. The obtained ROC AUC (0.70) was with 9.1% lower than the ROC AUC which was obtained in the present study (0.791). In addition, the PADR-EC Score did not evaluate the specific influence of all drugs on the hospitalization risk^16^. This limitation was solved in the present study, by implementing the machine learning classifier which considered the patient with regards to his complete pharmacological treatment. Certain ADR ranking was previously applied^15^; however, the ranking process was used for evaluating only the severity of the analyzed ADRs. Furthermore, it did not take into account patient related aspects besides genes-severe ADRs associations^15^. The sorted ADR terms could help in the patient safety monitoring, by allowing to consider the most relevant ADRs and differentiate them from clinical aspects claimed by the patient.

In the presented example, the list contains the first 20 relevant ADRs leading to serious conditions (which might cause initial, prolonged hospitalization, death or which might represent a life threat), such as “Hypokalaemia” (rank 1–10.91% probability of both appearing and leading to a serious event), “Hypotension” (rank 7, 4.92%), “Vomiting” (rank 8, 3.21%) “Renal failure” (rank 13, 1.09%) or Stevens-Johnson syndrome (rank 17, 0.09%). The obtained Total Score (51.91%) indicates a relatively high risk of developing severe ADRs (with a Severity Score equal or greater than 0.7). The Hospitalization Risk was 47.21%, while the Mortality Risk was lower, of 22.18%, both being very important ADR severity indicators which could be safely used in clinical practice. Using the SOC plot and the Total Score (stratified by using the Hospitalization and Mortality risks), the physician or clinical pharmacist could retrieve complex and critical aspects regarding the overall ADR profile, by allowing the comparison between two drug therapies for a certain patient. The SOC plot has the advantage of allowing the user to easily and rapidly differentiate the most important ADR groups related to the MedDRA SOC classification and therefore would be a very simple to use option both for out-patients and in-patients safety monitoring.

Regarding the predicted ADRs for the presented FAERS case, the application accurately predicted two of the three ADRs which lead to the patient’s hospitalization and death, vomiting (rank 8) and renal failure (rank 13). The septic shock could not be predicted by the patient-tailored algorithm, since it is not included in SIDER for either of the 8 drugs from the patient’s medication regimen. However, while analyzing the obtained SOC plot, besides the Gastrointestinal disorders (20.10% probability of leading to a serious event) and the Metabolism and nutrition disorders (17.67%), the program also found the Infections and infestations group (12.92%) as being a clinically relevant ADR class, which could be also correlated with the risk of developing septic shock, after a proper analysis of the results by a healthcare professional. In addition, in this specific case, the application correctly predicted the overall ADR severity, since a Total Score of 51.91% meant a high risk that the patient would either be hospitalized or will die due do ADRs.

Another advantage of the current application is the ability of performing selective stratifications based on the specific predictive variables. For example, in the presented real patient case, only a little decrease in the patient’s age (from 85 to 80) would imply a reduction of the total risk from 51.91% to 46.29%, while the same age increase would yield a total risk of developing severe ADRs of 54.98%. It should also be noted that the gender had also a significant influence on the results returned by the application in this specific case, since a female gender would have returned a lower risk of developing severe ADRs (42.55%, as compared to 51.91%). In addition, the web application has also the ability of returning stratified results on the basis of the drug therapy. In the current example, by replacing the selective beta blocker bisoprolol with carvedilol, a non-selective beta blocker with associated alpha-adrenolytic effect, the Total Score consistently escalated from 51.91% to 75.67%, with an increase in both hospitalization (from 47.21% to 69.57%) and mortality (from 22.18% to 41.84%) risks. The detailed results reflect the property of the program of performing relevant comparisons depending on the cumulative risk scores (Hospitalization Risk, Mortality Risk, Total Risk).

Due to its implemented machine learning algorithm and complex databases, the present application could also be used to perform relevant comparisons between two or more in-patients or out-patients. For example, when confronting the results obtained for the already presented patient case with another case from FAERS (a 45-year-old female patient suffering from multiple sclerosis and depression and receiving fingolimod, pregabalin and escitalopram), the algorithm accurately predicted a much lower risk of developing severe adverse events associated with the latter, more specifically a 2.40% hospitalization risk and 1.26% mortality risk (3.01% Total Score). Another analyzed case, that of a 76-year-old male patient suffering from arrhythmia (thrombosis risk factor) and receiving amiodarone, propranolol, clopidogrel, atorvastatin and midodrine, yielded a total risk of 45.88% of developing severe ADRs. The patient was hospitalized, but did not die due to the reported ADRs, which was quite accurately predicted by the machine learning algorithm, which returned a 41.27% hospitalization risk and a 19.29% mortality risk. The reported ADRs for this patient were atrioventricular block and sinoatrial block. According to the Drugs.com Interaction Checker, a very powerful online tool for such purposes, the interaction between propranolol and amiodarone, as well as the one between propranolol and midodrine can lead to serious cardiac rhythm disturbances, due to the additive effects which propranolol and amiodarone have on atrioventricular conduction. Secondly, midodrine, due to its vasoconstrictor effect, can lead to reflex bradycardia^14^.

The severity of these interactions was also assessed by the neural networks algorithm, which predicted the following relevant ADRs: bradycardia (3.995%, rank 6) and ventricular fibrillation (1.19%, rank 16). The higher risk which was attributed to bradycardia (3.995%) can be explained by the fact that this ADR can be developed due to both serious interactions (propranolol-amiodarone and propranolol-midodrine), as opposed to ventricular fibrillation (1.19%, one causal interaction – propranolol-amiodarone). The obtained results are of an utmost importance, since they prove the ability of the developed web application of offering a more detailed and personalized ADR risk as compared to Drugs.com Interactions Checker in terms of the overall ADR profile, as well as ADR groups and ADR terms stratification. Drugs.com Interactions Checker only classifies drug pairs interactions in minor, moderate or major^14^.

The total risk was also well estimated in comparison with the first detailed patient case, which had a higher risk of developing severe ADRs (51.91% Total Score) and resulted in both hospitalization and death due to the reported ADRs. Hence, by performing such analyses, the currently developed web application could have an essential role as a clinical tool during patient hospital admission, by offering the clinician the possibility of prioritizing the patients based on their specific risk of developing severe adverse events, after obtaining the specific ADR severity indicators.

Nevertheless, the developed ADR computer application prototype has a few limitations.

First of all, it does not take into account the drug doses and treatment duration. The doses and dosage regimen can play an important role in the development of ADRs^22^. However, no standardized DoTS classification is available for all ADRs, which yields a huge drawback in the possibility of quantifying them based on the dosage criteria; in addition, the FAERS quarterly files specify the drug dosage only in a limited amount of cases^19^.

A validation of the performances of the application was performed by evaluating the MLP classifier used in the Hospitalization and Mortality Prediction Models, which gave good results using the data from real patients from FAERS, yielding a 79.17% ROC AUC score (for Hospitalization) and an 85.4% score (for Mortality). The results obtained during the validation of the classification task also yielded satisfactory values regarding the computed metrics, with a F_1_ score higher than 75% for both the hospitalization and mortality prediction algorithms. The obtained precision (78.57% for hospitalization and 79.58% for mortality), which took into account the false positives, yielded higher values than the computed recall (73.01% for hospitalization and 71.95% for mortality), which took into account the false negatives. The overall lower recall values can be explained by the slightly imbalanced dataset, especially for the mortality prediction model, where a 1:4 ratio was found between the positive and negative class, which would mean that the algorithm might tend to be slightly biased towards classifying the observations as belonging to the negative class. However, the differences between precision and recall are not big (6–8%) and hence, this disadvantage cannot be considered significant, nor cannot it negatively influence the clinical relevance of the application, especially considering that the computed probabilities (as quantified by the ROC AUC Score, Brier Score and the reliability curve) were found to be well calibrated^23^. In addition, the web application is created in a manner that will allow future optimizations of both the included FAERS data and the machine learning algorithm, which might further increase the clinical relevance, as well as the statistical reliability of the model.

Despite the fact that the model included a relevant list of patient comorbidities, it did not include all the relevant personalized criteria, such as ethnicity or genetic polymorphisms, not embedded in the FDA reporting system^19^. For example, the Chinese and Japanese populations have an increased systemic exposure to Rosuvastatin, with a 2-fold increase in the AUC as compared to the Caucasians^24^.

In terms of interactions, the application embedded by default the pharmacodynamic drug interactions, due to the cumulative consideration of the drugs from the list which could cause a condition of interest, also reflected in the Severity prediction model. However, the pharmacokinetic drug interactions (PKDI) are not considered. For example, a study concluded that the concomitant use of Ciclosporin and Rosuvastatin (10 mg once daily) leads to a 7.1-fold increase in the AUC of Rosuvastatin, with a very high risk of liver damage and myopathy^24^. Another classical example is the PKDI between Omeprazole and Clopidogrel, which inhibits the formation of the active metabolite of Clopidogrel and therefore might lead to a certain reduction of the risk of developing ADRs which are related to the antiplatelet effect^25^.

The current manuscript is an optimized version of the 6^th^ Chapter of the PhD Thesis of the main author^26^. Several important issues present in the PhD Thesis were solved in the current article: the number of drugs was increased from 16 to 734 (by using the SIDER database, a standardized dataset of ADRs) and the mean ROC AUC Score was increased from 64.15–70.51% to 79.17–85.4%. Another optimization which was included was the use of FAERS system, which unlike the Eudravigilance system also includes the complete list of drug indications for a specific case and hence several important severity predictors could be added in the machine learning model^19^. In addition, an online platform was created and hence the application is available for free at www.adrriskestimator.com.

The presented characteristics of the current ADR application prototype reflect the potential of the program to be considered a useful tool within the personalized ADR resources currently available in healthcare. While a few such resources exist, as presented and discussed in the article, no models have yet been developed which could be able to individually analyze each relevant ADR for a certain patient and drug combination. The Statin Intolerance App developed by the American College of Cardiology (^13^) performs a very accurate and complex analysis of individual ADR terms, with the consideration of all relevant personalized criteria, but is only designed to analyze the musculoskeletal symptoms. Considering all the discussed advantages and drawbacks of the presented application, the long-term goal would be the inclusion in the model of all drug- and patient-related criteria which could influence the development of ADRs, in terms of both frequency and severity, as well as the computation of a personalized Frequency Score, optimizing the relevance of the ADR stratification process. After performing these additional steps, the application could be used as a professional tool in clinics, hospitals and community pharmacies, with a high clinical relevance.

## Conclusion

A web application prototype which allows the user to examine the most relevant ADRs which are likely to appear in a given drug therapy for a specific patient was designed and implemented. The application was based on three databases (SIDER, FAERS and MedDRA) and a Frequency Score and Severity Score were devised: the first based on the ADR incidence and the second – on the probability of a severe event (hospitalization or death) linked to the analyzed ADR. The application’s options, combined with the created scoring system allow the user to analyze the possible ADRs from different points of view, including a graphical analysis and a quantification of the obtained ADR ranks. These complex features could be used in an ADR personalized prediction, but also in the patient safety monitoring or therapy choice.

## Methods

### Selected drugs and databases

734 drugs and their ADRs were extracted from SIDER Database, considering the drug name, ADR name and frequency^17^. A total number of 4047 ADRs were selected, considering their Preferred Term (PT) according to the Medical Dictionary for Regulatory Activities (MedDRA). In addition, the System Organ Class (SOC) of each ADR was extracted from MedDRA, in order to provide additional information regarding the given ADR. ADR name harmonization was also performed, in order to avoid the situation in which an ADR term with the same meaning has different names for two different pharmacological agents^17,18^. A number of 60 harmonizations were performed, leading to an enhanced and relevant similarity degree within the adverse effects extracted from SIDER.

FAERS quarterly files contain detailed information regarding the spontaneous reports received by the Food and Drug Administration (FDA). FAERS was considered as source of information for the detailed spontaneous reports because the data contain the complete list of drug indications for each patient, leading to a coherent pathology list which might influence the development of ADRs^19^. A total number of 82681 reports containing ADR reporting information for the 734 drugs were selected, considering for each case the patient age and gender, drug list, drug indications, ADR names, hospitalization outcome (NO or YES – initial or prolonged hospitalization due to the ADR) and whether the reported ADRs lead to death or where marked as life-threatening (NO or YES – death or life threat). All the cases contain data obtained from real patients. The 82681 reports were extracted after eliminating from the dataset all the cases with unknown age, gender, drug indication, hospitalization outcome and/or data regarding mortality. The data were imported in Python programming language version 3.5.2. The FAERS information was used for building a prediction model of the severity due to the ADRs. The Hospitalization and Mortality fields were used as output variables, while the predictor variables were age, gender, specific drugs, ADR and specific drug indications (pathologies). From the 4047 ADRs included in SIDER, 3194 were also found in FAERS, leading to a high degree of terminology similarity between the two sources of information. A total number of 6 pathologies were included in the model, by considering the scientific available data regarding the comorbidities linked to a high risk of ADRs, as well as a recursive feature elimination algorithm performed in Python. The following pathologies were included as predictive variables: Renal insufficiency, Hepatic insufficiency, Diabetes mellitus, Hypertension, Cardiac failure and thrombosis risk factors (such as smoking or hyperlipidaemia)^7^. Using a Neural Networks (Multi-Layer Perceptron, MLP) machine learning classifier, the model predicted the probability that a patient with certain characteristics and given ADR(s) will be hospitalized or will die due to the specified ADR(s) in case the ADR appears. The two types of probabilities were combined in a final Severity Score. The personalized hospitalization and mortality prediction algorithms were embedded in the Scoring System development^23,27,28^.

### Data processing and scoring system

The Frequency Score was expressed as a probability and was computed based on the ADR average frequency percentage, derived from SIDER, by means of the probability mass function from a Poisson binomial distribution^29^, according to Eq. (1).

1 $$Frequency\,Score=1-\mathop{\prod }\limits_{i=1}^{n}(1-Frequenc{y}_{drug[i]})$$

where Frequency_drug[i]_ represents the ADR Frequency value of the i^th^ drug from the combination for which the analyzed ADR is present in its SIDER ADR list^29^.

The Severity Score was the patient-tailored part of the Scoring System development and reflects the probability that the patient will experience a severe event (hospitalization, life threat or death) in case the ADR appears. The Severity Score was computed based on the predicted probabilities obtained from the MLP classifier, according to Eq. (2).

2 $$\begin{array}{rcl}Severity\,Score(ADR[j]) & = & 1-(1-{\rm{Prhospitalization}}(ADR[j]))\\  &  & \ast \,(1-{\rm{Prmortality}}(ADR[j]))\end{array}$$

where Pr_hospitalization_(ADR[j]) and Pr_mortality_(ADR[j]) represent the Hospitalization Probability Score and the Mortality Probability Score computed for a certain patient for the j^th^ unique ADR by implementing the MLP algorithm.

The final MLP model which was built and which is currently available on the web platform (for both the hospitalization and mortality prediction) was an ensemble-type algorithm and consisted of five different MLP classifiers, each of them having the following hyperparameters: hidden layer size: 40, maximum number of iterations: 500, tolerance: 0.1 and logistic activation (which is appropriate for probability estimation). In addition, to account for the imbalanced data, an oversampling technique was implemented for the training set of the mortality prediction model, using the following weights: class 0: 52567, class 1: 57000, where 0 was the majority class (negative class – no death/life threat) and 1 the minority class (positive class death or life threat). These weights were set due to the fact that the training set (65694 patients) contained 52567 patients belonging to the negative class (no death/life threat) and 13127 belonging to the positive class (death/life threat). The random repetitive oversampling technique available in imblearn’s RandomOverSampler was used. The random state was set by default to the one available in numpy.random. The positive examples (minority class) were randomly oversampled in order to obtain a balanced dataset. The oversampling was performed in such a manner in order to reduce the bias and variance of the classifier^23^. For the hospitalization prediction, the dataset was much more balanced between the negative (no hospitalization) and the positive class (initial and/or prolonged hospitalization) and as a consequence, the standard class weighting available in sklearn’s MLP Classifier was used^23^.

Therefore, the final Severity Score will express the probability that the patient will be hospitalized or will die in case he develops the specified ADR.

A Total Score was also computed after the Frequency and Severity Scores were obtained. The Total Score was developed per patient and reflects the cumulative risk of developing serious ADRs with high risk of hospitalization for a specific patient with a given drug combination. In terms of ADR prediction, it quantifies the probability that a patient will develop at least one ADR related to a severe event from the ADRs with a Severity Score equal or greater than 70%. The Total Score was computed based on Eq. 3.

3 $$Total\,Score=1-\mathop{\prod }\limits_{j=1}^{Sev\ge 0.7}(1-Score[j])$$

where Score [j] represents the product between Frequency[j] and Severity [j] (the Frequency and Severity Probability Scores computed for the j^th^ ADR from the combination)^29^. It should be noted that the Total Score is obtained by considering only the ADRs with a higher degree of severity, namely the ones with a Severity Score greater than 0.7. The ADRs with a Severity greater than 0.7 are further sorted on the basis of their score (Frequency x Severity) and are listed according to their specific rank (the ADR with the highest Score will receive a rank of 1, the second ADR - rank 2, and so on). Therefore, due to the algorithm by which the ADRs are sorted, the program will return the most relevant adverse reaction in terms of a combination between frequency and severity. The scoring and ranking system was built in this manner in order to offer a simple, yet cumulative and clinically relevant approach on the most important ADRs which might appear for a specific patient with a given drug therapy.

In addition to the Total Score, a SOC Score was also devised, by implementing the same formula on each SOC grouping (example: Gastrointestinal disorders, Cardiac disorders). The SOC Score was further used in the application in order to allow a differentiation between the most important ADR groups according to their SOC. Since they are computed as probabilities, the Frequency, Severity, Total and SOC Scores range between 0 and 1. In addition, the causal relation between the drugs and the specific ADR terms was obtained by combining the information from SIDER with the machine learning prediction model (which is able to establish correlations between predictive variables) and by eliminating the ADRs from FAERS which were not found in SIDER^17,19,23^.

### Severity score validation

The application performances were evaluated by performing a validation on the severity prediction in terms of different statistical measures. Both the output class of the predictor and the output class probability were validated for both hospitalization and mortality.

The predictors were evaluated by output class using the following measures: accuracy, precision, recall and F1 score. Accuracy took into consideration the percentage of correctly classified instances (one instance represented a triad: patient (age, gender, pathologies-drugs combination-ADR), the precision evaluated the ability of the model to avoid the false positive (FP) results, while the recall was inversely proportional to the number of false negatives (FNs). For example, the lower the number of FPs is, the higher is the precision value. The F1 score was computed as a weighted average between precision and recall and presented the predictor’s overall performance. Additionally, the Brier score was used to evaluate the estimated class probability, by computing the average squared difference between the predicted probabilities of belonging to class 1 and the actual class (0 or 1). The lower the Brier score value is, the more reliable is the probability prediction. Another important measure for evaluating the overall performance of the model in terms of probability estimates was the Area Under the Receiver Operating Characteristic Curve Score (ROC AUC score). This was the most important validation parameter, since it measured discrimination i.e. the ability of the predictor to correctly classify the cases with both present hospitalization which didn’t require hospitalization. The computation of the score was based on the plot of True Positives versus False Positives. Basically, an ideal ROC AUC will mean that x% of the cases with x% hospitalization probability predicted by the classifier actually required hospitalization (class 1). The estimation of how well calibrated the predicted probabilities are was performed by computing a reliability curve^23^.

The mortality prediction model was evaluated in an identical manner.

Table 2 briefly presents the computation of the validation measures, as well as their ranges and optimal values. Four machine learning classifiers from Python sklearn library were compared by using these computed measures, i.e. K-Nearest Neighbors, Random Forest Classifier, Multi-Layer Perceptron (MLP) (a type of Neural Networks) and Logistic Regression. The dataset was divided in two and a hold-out validation was performed, due to the large size of the data. The training set was represented by 65694 patients and the test set was represented by 16987 patients. The validation measures estimated the results obtained on the test set. MLP was finally chosen for building the severity prediction model, due to the good balance between the computed measures and the reliable probability estimates evaluated by the ROC AUC and Brier scores^23,27,28^. Therefore, the MLP model will estimate the probability that a certain patient will experience a distressing event which leads to initial/prolonged hospitalization, death (or which represents a life threat) due to the ADR in case the ADR appears.

**Table 2:** Computation of the validation measures, ranges and optimal values^23,26,27^.

Validation measure	Formula	Range	Optimal value
Accuracy	$\frac{Number\,of\,correctly\,classified}{Total\,number\,of\,instances}$	0–100%	100%
Precision	$\frac{TP}{TP+FP}$	0–100%	100%
Recall	$\frac{TP}{TP+FN}$	0–100%	100%
F1 score	$\frac{2\times Precision\times Recall}{Precision+Recall}$	0–100%	100%
Brier score	$\frac{1}{N}x\mathop{\sum }\limits_{1}^{N}{(Pr(1)-Class)}^{2}\ast$	0–1	0
ROC AUC score	Based on the TP rate (recall) vs. FP rate graphical representation	0–100%	100%

^*^N = the total number of instances, Pr (1) = the predicted probability of belonging to class 1 (initial or prolonged hospitalization due to the ADR(s), Class = the true class (0 or 1).

### Graphical user interface and application development

The application development was based on the creation of an online platform (available at www.adrriskestimator.com), allowing the introduction of data (drugs, age, gender, pathologies) and the presentation of the outputs. Flask was used as module for the application development process and Python Anywhere was used as online tool^30^.

All the unique ADR terms were extracted from SIDER database and were also used as inputs for computing the scores. The Scoring System was thereafter implemented, as a measure for the ADRs ranking. Thereafter, the outputs are presented as sorted lists and specific bar plot representations.

All the options and specific outputs of the application are detailed in Table 3.

**Table 3:** The output options and details.

Output	Output details
Total Score	A horizontal bar plot representation of the Total Score (developed per patient), as well as a stratification based on the total Hospitalization Risk and Mortality Risk
SOC plot	A bar plot representation of the first 5 SOC groupings in terms of their SOC Score. The ADRs with a Severity Score equal or greater than 0.7 are considered. The classes are sorted in descending order. Allows SOC groupings risk stratification.
ADR sorted list	Returns a list, where each ADR receives a specific rank based on its Score (Frequency Score x Severity Score – the probability that the ADR will appear and will lead to a serious event: hospitalization or death). The list is sorted in descending order of the selected score. The ADRs with a Severity Score equal to or greater than 0.7 are considered.

### Ethics and biosecurity

This article does not contain any studies with human participants or animals performed by any of the authors.

The study only involved the analysis of patient data collected by FDA through FAERS. The information did not include any personal data, in accordance to the data protection legislation, nor did it involve patient counselling. Therefore, informed consent was not required and no ethics approval was needed (this complies with national guidelines)^31,32^. In addition, no identifiable information was included in the manuscript. No permissions were required to access the patient data used in the study.

## Data Availability

The application is available online at www.adrriskestimator.com.
