# Author Correction: The development of a scoring and ranking strategy for a patient-tailored adverse drug reaction prediction in polypharmacy

**DOI:** 10.1038/s41598-021-85551-5

**Published:** 2021-03-09

**Authors:** Andrei Valeanu, Cristian Damian, Cristina Daniela Marineci, Simona Negres

**Affiliations:** 1 Carol Davila University of Medicine and Pharmacy, Faculty of Pharmacy, Department of Pharmacology and Clinical Pharmacy, 6 Traian Vuia St., 020956 Bucharest, Romania; 2 Polytechnic University, CEOSpaceTech, 7 Gheorghe Polizu St., Building P, 1st. floor, 011061 Bucharest, Romania

Correction to: *Scientific Reports* 10.1038/s41598-020-66611-8, published online 12 June 2020

The Acknowledgements section in this Article was omitted. The Acknowledgements section should read:

“The work has been funded by the Operational Programme Human Capital of the Ministry of European Funds through the Financial Agreement 51675/09.07.2019, SMIS code 125125.”

